# MultiPhys: Heterogeneous Fusion of Mamba and Transformer for Video-Based Multi-Task Physiological Measurement

**DOI:** 10.3390/s25010100

**Published:** 2024-12-27

**Authors:** Chaoyang Huo, Pengbo Yin, Bo Fu

**Affiliations:** School of Mechanical Engineering, Sichuan University, Chengdu 610065, China; huochaoyang@stu.scu.edu.cn (C.H.); yinpengbo@stu.scu.edu.cn (P.Y.)

**Keywords:** remote photoplethysmography, multi-task physiological measurement, network fusion, deep learning

## Abstract

Due to its non-contact characteristics, remote photoplethysmography (rPPG) has attracted widespread attention in recent years, and has been widely applied for remote physiological measurements. However, most of the existing rPPG models are unable to estimate multiple physiological signals simultaneously, and the performance of the limited available multi-task models is also restricted due to their single-model architectures. To address the above problems, this study proposes MultiPhys, adopting a heterogeneous network fusion approach for its development. Specifically, a Convolutional Neural Network (CNN) is used to quickly extract local features in the early stage, a transformer captures global context and long-distance dependencies, and Mamba is used to compensate for the transformer’s deficiencies, reducing the computational complexity and improving the accuracy of the model. Additionally, a gate is utilized for feature selection, which classifies the features of different physiological indicators. Finally, physiological indicators are estimated after passing features to each task-related head. Experiments on three datasets show that MultiPhys has superior performance in handling multiple tasks. The results of cross-dataset and hyper-parameter sensitivity tests also verify its generalization ability and robustness, respectively. MultiPhys can be considered as an effective solution for remote physiological estimation, thus promoting the development of this field.

## 1. Introduction

In recent years, with the collaborative development of computer science and biomedicine, physiological monitoring has attracted widespread attention and has been applied in various fields [1,2,3,4]. It is necessary to extract relevant indicators for physiological monitoring, such as Blood Volume Pulse (BVP), Heart Rate (HR), Heart Rate Variability (HRV), Respiration Rate (RR), and Blood oxygen Saturation (SpO2).However, previous measurements, such as Electrocardiography (ECG) and Photoplethysmography (PPG), are needed when performing measurements in direct contact with human skin, leading to inconvenience and discomfort for patients requiring long-term physiological monitoring or daily measurements for healthcare purposes [5]. To address this issue, the rPPG method, which has the characteristics of remote monitoring and can transform ubiquitous RGB cameras into medical care sensors [6,7,8], has been widely used in the field of remote physiological measurement. For example, most sports bracelets or watches currently on the market use this method to extract daily health indicators from the human body [9]. However, rPPG methods are not perfect either. Existing rPPG methods mainly extract physiological signals by measuring changes in light absorption and scattering [10,11]; however, as the rPPG signal is very weak, noise such as environmental light and motion can cause significant interference of the signal [6,7,8].

To achieve robustness in complex environments, deep learning approaches have been widely applied for rPPG [12,13,14,15]. Among them, CNN and transformer models are the most popular, which mainly focus on one prediction task (e.g., BVP or RR) [11,16]. In addition, some studies have also attempted to measure different physiological signals simultaneously using a shared representation [17] or the temporal shift module [18]. However, there are still some deficiencies; for example, the receptive fields of CNNs [19,20] are limited and, from the perspective of computational complexity, transformer-based [21] architectures perform poorly in capturing long-term dependencies, especially when dealing with long video sequences [22].

The Mamba architecture [23] has recently received extensive attention. While the powerful attention mechanism of Transformer enables it to focus on information at each position of the sequence simultaneously, it consumes a large amount of computing resources. In contrast, Mamba, with its Selective State-Space Model (SSM), reduces the quadratic computational complexity of the Transformer to linear complexity, and can capture long-term dependencies at the same time.

Inspired by the above discussion, we studied different integrations of heterogeneous networks with different advantages in order to propose a fusion model for multi-physiological indicator measurement. The proposed MultiPhys model is composed of a feature extraction part and a feature selection part. In particular, we adopted a hierarchical architecture as the feature extraction part, first using CNN-based layers to quickly extract features. Then, we improve the Mamba integrator block to compensate for the content lost due to the sequential constraints of the SSMs. After passing through the Mamba integrator block, the attention layer compensates for the insufficient receptive field of the CNN layer and significantly enhances the global context and long-range spatial dependencies. Furthermore, for the feature selection part, we set up a gate block to receive the previously generated fused physiological features, and dynamically select element-level features for different tasks through the sigmoid function. Finally, the task-specific signals are estimated by different heads based on corresponding selected feature vectors.

The main contributions of this study are as follows:

- We adopt the heterogeneous fusion design principle. Specifically, we integrate the CNN, Transformer, and Mamba models, which enable our MultiPhys to combine the linear computational complexity of Mamba, the ability of Transformer to capture global context and long-distance dependencies, and the power of CNN to quickly extract local features, thus enhancing the model’s feature extraction ability.
- We propose a combination of gating and feature extraction structures. The gating mechanism aims to enables more targeted selection of the low-level features extracted from shared backbone modules for different physiological signal outputs.
- Extensive experiments prove the superior performance of our model in the context of multi-task physiological measurement. The cross-dataset test and hyper-parameter sensitivity test further demonstrate the generalization ability and robustness of our model, respectively.

## 2. Related Work

### 2.1. Non-Contact Physiological Measurement

In the development of physiological measurement methods, rPPG has widely replaced the past contact-based physiological measurements due to its non-contact nature. In the past, rPPG approaches mainly adopted the method of signal processing to extract periodic signals through the analysis of facial videos [24,25,26,27]; however, the results were easily interfered with noise in complex environments, and these methods were gradually replaced by others based on deep learning. A large number of deep learning methods have adopted the CNN and transformer architectures as the backbone network [28,29]. The CNN architecture has been continuously optimized, transitioning from 2D CNNs [18,19,30,31] to 3D CNNs [20,32,33], which improves their accuracy through the introduction of temporal data [20]. However, 3D CNNs also lead to more computations and parameters. Later, ref. [34] proposed the use of the temporal shift module, which moves the tensor along the temporal dimension to promote the exchange of information between multiple frames. This optimization not only improves the accuracy, but also only requires the computational cost of 2D CNNs; however, this method does not perform well when attempting to capture long-range dependencies. Transformer-based methods, which use attention mechanisms, can ignore the noise unrelated to the extracted information in the video and can understand the periodic pattern of rPPG in the long-term context environment; however, the quadratic computational complexity of the transformer leads to higher computational consumption [35,36,37].

In order to take advantage of both architectures, which are able to extract subtle rPPG signals from video clips with a large amount of spatio-temporal redundancy and understand the periodic pattern of rPPG in the long-term context environment, combining convolution operations with the attention mechanism module has become a new direction in deep learning research. Recently, ref. [38] proposed a fully end-to-end model, called RhythmMamba, for remote physiological measurement. It extracts rPPG features in the skin area using an attention mechanism and performs feature extraction and information integration using a CNN architecture, improving the ability of the model to measure and analyze remote physiological signals; however, this model mainly focuses on a single task.

### 2.2. rPPG-Based Multi-Task Physiological Measurement

Multi-task learning presents remarkable advantages in scenarios involving multiple related tasks [39,40,41], such as the rPPG multi-task problem. Through training different tasks in parallel and utilizing shared representations, the intrinsic connections among tasks can be effectively explored, thus enhancing the generalization ability of the model [42]. Many studies have integrated multi-task learning with deep learning to overcome the difficulties of rPPG multi-task problems. This method prompts the model to learn related information between different tasks, which significantly improves the accuracy of the estimation [43,44]. For example, BigSmall has a unique multi-task model architecture that includes a high-resolution spatial branch and a low-resolution temporal branch. This design ingeniously utilizes differences in spatial and temporal scales, greatly reducing the computational load. At the same time, it uses the wrapped temporal shift module to successfully achieve efficient multi-task learning for tasks with different spatio–temporal characteristics such as facial actions, breathing, and pulse. When dealing with complex physiological signal-related tasks, this model can accurately capture the characteristics of different tasks in the spatio–temporal dimension and realize the collaborative optimization of multiple tasks through the learning of shared representations [17]. In addition, MTTS-CAN uses the loss function of pulse and respiration signals to simultaneously train the estimation tasks of two physiological indicators, solving the challenging problem of multi-task non-contact cardiovascular signal measurement [18]. All of the above methods perform prediction and estimation of physiological signals by learning from facial video to generate shared representations. This is based on the fact that the two physiological signals from the same facial area have significant correlations [45]. This mode fully utilizes the dependencies among various indicators, significantly reducing the computational cost while improving efficiency. Through extensive research, it has been found that cardiovascular and respiratory measurements can be performed simultaneously. The features of facial movement, breathing, and HR are closely related, and the features of SpO2 are also closely related to the features of HR and RR [17,18,46]. However, previous multi-task models have certain limitations in generating shared representations and have not fully considered integrating the advantages of architectures such as CNN, transformer, and Mamba to further improve their efficiency. We hope to improve the efficiency and generalizability of the model through our proposal.

## 3. Method

### 3.1. Problem Formulation

This study provides a summary and the definitions of key symbols in Table 1. We first compress continuous facial videos into the spatial–temporal map (STMap) format [47]. To enhance the generalization ability of the model, we augment the STMap through color jitter and blur augmentation [48], and utilize the result as the input X. Then, X is input into the MultiPhys model, whose size is H×W×3 (where *H*, *W*, and 3 denote height, width, and the number of channels, respectively). In our work, the final prediction result is the four vital signs of the subject $\hat{Y}=\left\{{\hat{y}}_{HR},{\hat{y}}_{BVP},{\hat{y}}_{SpO2},{\hat{y}}_{RR}\right\}$, representing heart rate (HR), blood volume pulse wave of blood pressure (BVP), blood oxygen saturation (SpO2), and respiratory rate (RR), respectively. The formula of our model is expressed as $F\left(X\right)=\hat{Y}$.

In previous studies, different physiological indicators have been shown to be influenced by the same physiological movement and, so, there are certain correlations between different physiological indicators. For example, both HR and BVP are influenced by cardiac activity [49]. The oxygenation level affects the light absorption characteristics of tissues, manifested as SpO2 affecting BVP [50]. The proposed MultiPhys, based on traditional CNN, transformer, and Mamba architectures, realizes full understanding of the global context first and then makes accurate predictions for local regions. This method is more suitable for our visual physiological prediction tasks, thereby enhancing the ability to capture signals related to the above-mentioned physiological indicators in images.

### 3.2. MultiPhys

**Figure 1:**
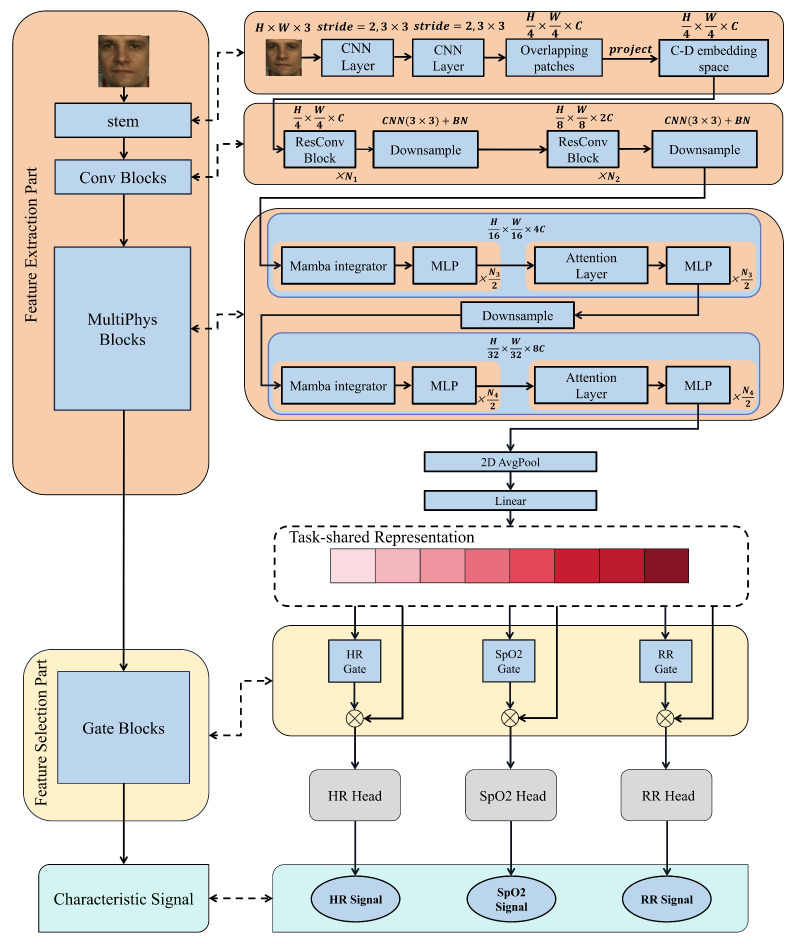
The overall architecture of proposed MultiPhys. The different red squares of Task-shared Representation represent different feature values after feature selection.

As shown in Figure 1, the MultiPhys model consists of two main parts. One is the Feature Extraction Part, which aims to quickly extract relevant signals from the image and generate a Task-shared Representation. The other is the Feature Selection Part, which follows the Feature Extraction Part. Different tasks will eventually predict multiple physiological signal values simultaneously through their respective gates. Discussions of each section are presented below.

#### 3.2.1. Feature Extraction Part

**Stem**—The Stem is composed of two consecutive 3×3 CNN Layers. When the input X $\in R^{H\times W\times 3}$ enters the Stem, it is first transformed into overlapping patches of size $\frac{H}{4}\times \frac{W}{4}\times C$. Subsequently, the overlapping patches will be projected into the embedding space of dimension *C*. This process quickly captures local and global information.

**Conv Blocks**—This stage is composed of layers based on Residual CNN, which can quickly extract features at a higher resolution. A fter every $n=\{N_{1},N_{2}\}$ CNN layers, a DownSampler is respectively connected, which consists of a 3×3 CNN layer with a stride of 2 and batch normalization. While reducing the resolution of the image by half, features at different levels are obtained. The ResConv Block is mainly composed of *n* Residual CNN layers, and its specific calculation formula is depicted in Equation (1). The entire Conv Blocks contains two ResConv Blocks and two Downsamplers.

(1) $$\begin{array}{cc} \hat{\alpha } & =GELU(BN\left(Conv_{3\times 3}\left(\alpha \right)\right)),\\ \alpha & =BN(Conv_{3\times 3}\left(\hat{\alpha })\right)+\alpha . \end{array}$$

In the above formula, α is the output of the previous CNN layer, which also serves as the input of the next CNN layer. GELU and BN denote the Gaussian Error Linear Unit activation function and batch normalization, respectively.

**MultiPhys Blocks**—In this stage, through introducing the redesigned Mamba integrator, MLP Blocks, and Attention layers, the model is able to efficiently process the visual information in computer images, recover more lost physiological information in the images, capture long-range dependencies, and maximize the integration of various physiological information in the images.

The structured state space model (S4) and its variants (e.g., Mamba) have distinguished themselves with a SSM layer and a selection mechanism through its selective scan operation. After the input enters the MultiPhys blocks, it is linearly projected and sliced into sequences. Then, they will pass through convolutional layers, activation functions, and SSM. The SSM can transform the one-dimensional continuous input ρ(t)∈R into φ(t)∈R through the learnable hidden state $\psi (t)\in R^{S}$. The SSM is modeled by the following Equation (2):

(2) $$\begin{array}{cc} \psi^{\prime }\left(t\right) & =A\psi (t)+B\rho (t),\\ \varphi (t) & =C\psi (t). \end{array}$$

In the above formula, the evolution matrix $A\in R^{S\times S}$, projection matrix $B\in R^{S\times 1}$, and projection matrix $C\in R^{1\times S}$. *S* represents the sequence length. To improve computational efficiency, SSM needs to discretize the above parameters [51] to obtain the discrete parameters $\bar{A}\in R^{S\times S}$, $\bar{B}\in R^{S\times 1}$, $\bar{C}\in R^{1\times S}$. The transformation is performed by employing the zero-order hold method:

(3) $$\begin{array}{cc} \bar{A} & =exp(\Delta A),\\ \bar{B} & ={\left(\Delta A\right)}^{-1}\left(exp(\Delta A)-I\right)\cdot \left(\Delta B\right),\\ \bar{C} & =C, \end{array}$$

where **I** represents the identity matrix and Δ is a time-scale parameter. Then, we can utilize the discrete parameters to express Equation (2) as:

(4) $$\begin{array}{cc} \psi (t) & =\bar{A}\psi \left(t-1\right)+\bar{B}\rho \left(t\right),\\ \varphi (t) & =\bar{C}\psi \left(t\right). \end{array}$$

In addition, for the input sequence of size *S*, we can effectively solve the obstruction of the above discrete form by its inherent sequential nature through the convolution operation. The specific process is shown in Equation (5), where $\bar{K}\in R^{S}$ denotes a structured convolution kernel and ∗ indicates a convolution operation.

(5) $$\begin{array}{cc} \bar{K} & =(C\bar{B},C\bar{\mathrm{AB}},\ldots ,C{\bar{A}}^{S-1}\bar{B}),\\ \varphi & =\rho \;*\;\bar{K}. \end{array}$$

Our Mamba integrator consists of the main path and the vice path. We used a regular Conv layer to replace the causal Conv layer in the conventional Mamba block, in order to avoid the unnecessary restrictions of causal convolution in visual tasks. The sequence passes through the linear layer and the convolution layer in sequence on the main path, and then undergoes a selective scan operation through SSM. Compared with the main path, the Vice path lacks the SSM part. The main purpose of setting the Vice path is to capture more global physiological information. Finally, we connect the two outputs of the sequence after passing through both paths and project it through a linear layer. Using the Mamba integrator, we obtain a richer representation of physiological features and an improvement in image processing performance. The specific process is shown in Equation (6):

(6) $$\begin{array}{cc} \beta_{main} & =Scan(SiLU\left(Conv\left(Linear\left(C,\frac{C}{2}\right)\left(\delta \right)\right)\right)),\\ \beta_{vice} & =SiLU(Conv\left(Linear\left(C,\frac{C}{2}\right)\left(\delta \right)\right)),\\ \beta_{MI} & =Linear\left(\frac{C}{2},C\right)\left(Concat\left(\beta_{main},\beta_{vice}\right)\right), \end{array}$$

where Scan represents the selective scan operation [23], SiLU is the abbreviation of Sigmoid Linear Unit [52] which is the activation function after the Conv layer, Concat represents the concatenation operation, and $Linear(C,\frac{C}{2})$ represents that the input dimension and output dimension are *C* and $\frac{C}{2}$, respectively. Furthermore, δ is the output of Conv Blocks; $\beta_{main}$ and $\beta_{vice}$ represent the outputs of the main path and the vice path, respectively; and $\beta_{MI}$ is the output of the Mamba integrator.

After passing through the Mamba integrator, $\beta_{MI}$ will enter the MLP layer. These two parts can be regarded as a whole. Assuming that the input is γ, the output of the $\tau_{th}$ layer of this whole can be calculated as:

(7) $$\begin{array}{cc} \hat{\gamma^{\tau }} & =Integrator\left(LN\left(\gamma^{\tau -1}\right)\right)+\gamma^{\tau -1},\\ \gamma^{\tau } & =MLP\left(LN\left(\hat{\gamma^{\tau }}\right)\right)+\hat{\gamma^{\tau }}. \end{array}$$

In Equation (7), LN and Integrator represent the layer normalization operation and the Mamba integrator operation to select different tokens, respectively. Assuming that a stage has *N* layers, then the first *N*/2 layers go through the above calculation process, and the remaining *N*/2 layers adopt our attention mechanism to restore global physiological data features and capture the long-distance dependency relationships of physiological signals in space. The specific steps are as follows:

(8) $$\begin{array}{cc} Attention(Q,K,V) & =\Gamma (\frac{QK^{T}}{\sqrt{h}}V). \end{array}$$

In Equation (8), Q,K,V, and *h* represent query, key, value, and the number of attention heads, respectively [53,54]. After the above steps, the output will pass through a sampler and then go through the same process as detailed in the above steps again, which improves the robustness and generalization ability of the model, reduces the chance of overfitting, and ensures that the model thoroughly processes the input. After completing the above steps, the features will undergo a 2D average pooling and be projected through a linear layer. Finally, the Task-shared representation is obtained, in preparation for entering the Feature selection Part to classify different physiological signals.

#### 3.2.2. Feature Selection Part

**Figure 2:**
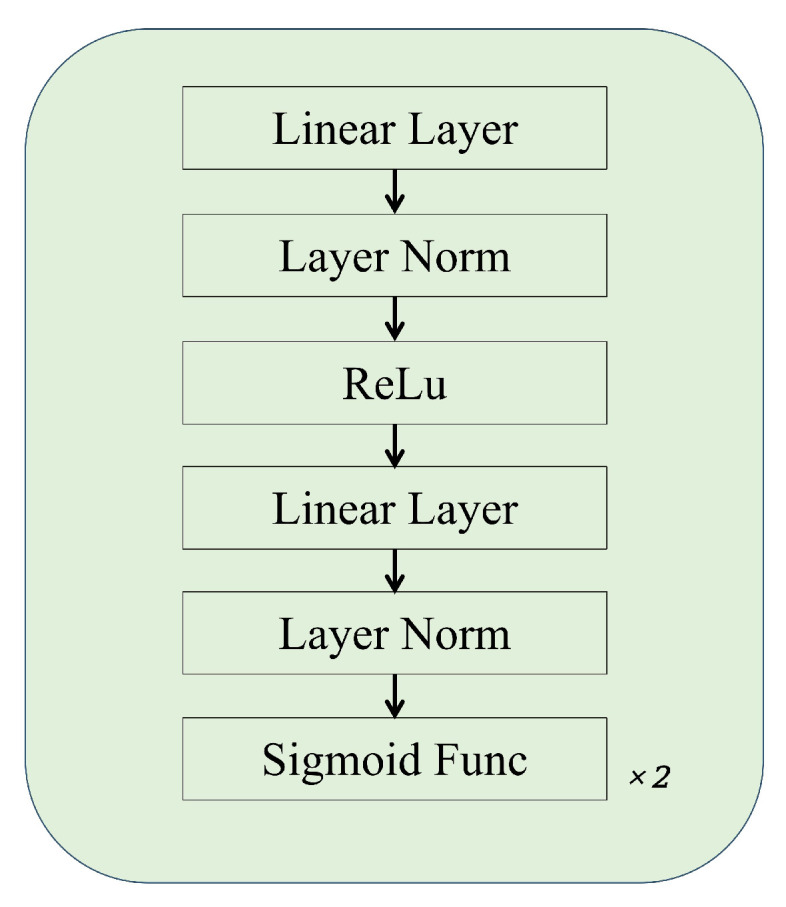
The architecture of gate.

**Gate Blocks**—The gate blocks classify the previously Task-shared representations. Through three branches, the predicted values of three groups of physiological signals are finally obtained. Our gate block is shown in Figure 2. The previous feature vector is first projected to a linear layer, following which layer normalization is performed to reduce the possibility of gradient explosion. Then, the ReLU function is used to increase nonlinear features. Through a linear layer and layer normalization again, the model better learns and fuses features at different levels. Finally, through two sigmoid functions, $\sigma =\{\sigma_{HR},\sigma_{SpO2},$ and $\sigma_{RR}\}$ (denoting the output of each gate) are obtained. Then, they are multiplied by the Task-shared representation *r*. In this way, we finally obtain the task-specific features $r^{\prime }=\left\{{r^{\prime }}_{HR},{r^{\prime }}_{SpO2},{r^{\prime }}_{RR}\right\}$. Finally, each $r^{\prime }$ is input into the corresponding estimation head to obtain the predicted physiological signals $\hat{Y}=\left\{{\hat{y}}_{HR},{\hat{y}}_{SpO2},{\hat{y}}_{RR}\right\}$.

### 3.3. Results and Loss Function

In addition to the loss function formulated above, in order to facilitate the estimation of different heart rates, we also formulate four loss functions; namely, $L_{HR}$, $L_{SpO2}$, $L_{RR}$, and $L_{BVP}$. The first three use the L1 loss to predict the average value of their respective signal distributions, while the last one uses the negative Pearson’s correlation coefficient for prediction. In addition, to suppress meaningless regularization in the early stage, we introduce an adaptation parameter θ [55]. Finally, we introduce the trade-off parameter $w_{i}$, and the overall loss function formulated as follows:

(9) $$\begin{array}{cc} L(X,\hat{Y}) & =\theta \left(w_{1}L_{SpO2}+w_{2}L_{RR}\right)+L_{HR}. \end{array}$$

## 4. Experiment

In our experiment, three physiological signals based on the physiological measurement of rPPG (i.e., HR, SpO2, and RR) were evaluated using three public domain datasets; namely, PURE [56], VIPL-HR [57], and V4V [58].

### 4.1. Datasets and Evaluation Metrics

In this study, we selected three different datasets, including various motion, camera, and lighting conditions, in order to evaluate the performance of our Mamba in simultaneously predicting physiological markers in rPPG. VIPL-HR [57] is a large-scale dataset of 2378 visible light videos and 752 near-infrared videos obtained from 107 subjects. The frame rate of the video varies from device to device. PURE [56] contains 60 RGB videos from 10 subjects recorded in 6 different settings. It also contains multiple vital signs (such as blood oxygen level), in addition to HR and BVP. This makes it highly suitable for multi-task training. The V4V [58] data present drastic changes in 367 physiological indicators and mainly simulate 10 tasks, but only HR is available and there is no BVP label. The first dataset consists of eight videos (approximately 16,500 frames), while the second dataset contains 42 RGB videos recorded using a Logitech C920 HD Pro webcam at 640 × 480 resolution and 30 FPS. We normalized the video and corresponding BVP signals of the three datasets to 30 Hz via cubic spline interpolation.

Based on existing similar methods [29,59], we evaluated HR, RR, and SpO2 measurements using the mean absolute error (MAE), root mean square error (RMSE), and Pearson’s correlation coefficient (P).

### 4.2. Baselines

To comprehensively evaluate the performance of MultiPhys in handling multiple tasks simultaneously, we selected several traditional rPPG single-task methods (GREEN [49], CHROM [24], POS [60], ARM-SpO2 [61], and ARM-RR [61]), deep learning-based rPPG single-task methods (RhythmNet [57], BVPNet [16], Dual-GAN [59], PhysFormer++ [21], EfficientPhys [37], rPPG-MAE [62], Contrast-Phys+ [63], rSPO [64]), and deep learning-based multi-task rPPG methods (MTTS-CAN [18], BigSmall [17], PhysMLE-R [47], PhysMLE-T [47]). Moreover, to offer more physiological monitoring models, we constructed one multi-task baseline based on ResNet18 [65] and another based on ViT-base [66]. Briefly, we utilized ResNet18 and ViT-base as the backbone networks to receive the STMap as input, then employed three different classification heads to directly carry out multi-task estimation. In addition, both video-based and STMap-based methods were taken into consideration. Among the methods referenced earlier, RhythmNet uses a GRU to aggregate the temporal information of multiple clips, which led to an unfair comparison. Therefore, we removed the GRU mechanism from RhvthmNet.

### 4.3. Implementation Details

Our model was implemented using the Pytorch framework. For pre-processing, following [57], we first used the open-source SeetaFace face detector to detect the face and locate 81 facial landmarks; then, we designed a face bounding box to remove the background area and align the face region. Our entire experiment was conducted on an RTX A6000. The batch size B and the number of iterations $N_{iter}$ were 60 and 20,000, respectively. We used the Adam optimizer for training, with a learning rate of 0.00001. In addition, in our experiment, we set the trade-off parameters $w_{1}$ to $w_{3}$ to 0.0001, according to the experimental results.

### 4.4. Multi-Task Comparison Experiments

#### 4.4.1. HR Estimation

**Figure 3:**
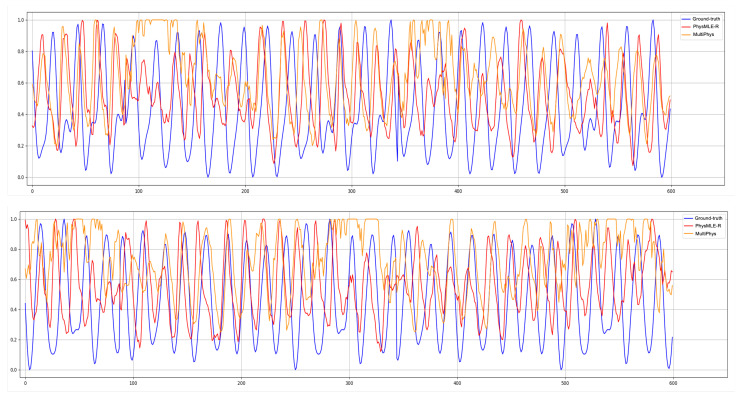
Comparison between the predicted BVP signal and the ground-truth BVP signal given two STMaps with 600 frames from PURE. The BLUE line indicates the ground-truth signal, the RED line is the signal by PhysMLE-R, and the ORANGE line is given by MultiPhys.

We presents the HR measurement results in Table 2, Table 3 and Table 4. Furthermore, the BVP signals output from our proposal and the SOTA baseline (i.e., PhysMLE [47]) are visualized and compared to the ground-truth in Figure 3. According to the results on the PURE dataset, the deep learning-based rPPG methods were superior to the traditional rPPG single-task methods, as the traditional methods are based on stricter assumptions and had not been trained with a large amount of data [62]. Deep learning-based methods can automatically learn features from large-scale data, and their performance usually increases with an increasing volume of data. Therefore, under the condition of large-scale data, the effect of the traditional methods was not as good as that of the deep learning-based methods. In addition, deep learning methods can automatically learn the multi-level abstract features of data, while traditional methods often rely on artificially designed features [67], which requires significant prior knowledge and experience regarding the domain, and still may not be able to fully capture the complex patterns in the data. Therefore, in a changing environment, the effect of the traditional methods is not as good as that of the deep learning-based methods.

In terms of trends, the performance of the deep learning-based multi-task methods was mostly better than that of the deep learning-based single-task methods. However, overall, the newly designed deep learning-based single-task methods (e.g., rPPG-MAE [62] and Contrast-Phys+ [63]) performed better than most of the deep learning-based multi-task methods, except for MultiPhys. This is because, although multi-task methods can utilize the correlations between different tasks—for example, the change of SpO2 being linked to the light absorption characteristics, thereby affecting the BVP signal, and then the HR is obtained by calculating the number of peaks in the BVP signal [68]; in addition, the change in RR will affect the autonomic nervous system, leading to a periodic change in heart rate [69]—the above correlations are often not causal and are unstable, leading the seesaw effect to inevitably occur during the training of multi-task models.

Next, referring to Figure 3, we notice that the BVP signals given by MultiPhys are more steady and smooth than those of PhysMLE-R. Meanwhile, as presented in Table 2, the MAE index of MultiPhys was about 45% better than that of ResNet18 [65], which mainly adopts a CNN architecture. This is because, compared with the traditional CNN architecture, our MultiPhys also incorporates the Transformer to capture global context relationships and long-distance dependencies. In addition, in order to reduce the computational complexity and improve the model’s accuracy, we added Mamba, which greatly improved its computational efficiency. Therefore, our MultiPhys makes up for the quadratic computational complexity of the traditional CNN architecture and enhances its ability to capture information, showing a significant advantage in the comparison of MAE. Furthermore, it performed about 47% better than ViT-base [66], which mainly adopts the transformer architecture. This optimization may come from the fact that, after the shared features in our model are learned through MultiPhys, these features are decoupled at the final estimation head through the gates of different tasks, and as the MultiPhys model can process visual information more efficiently. Compared with the ViT-base structure based on the transformer (which adopts hard parameter sharing), our MultiPhys model has a stronger ability to capture the subtle changes in the light cycle of the skin. Therefore, it can better handle the correlations between HR and other indicators, thus improving the performance of the model.

#### 4.4.2. SpO2 Estimation

As the V4V dataset does not contain sufficient information related to SpO2 measurement, we mainly used the PURE and VIPL-HR datasets to evaluate the performance of SpO2, and the test results are presented in Table 2 and Table 3. In addition, the measurement of SpO2 is closely related to the light absorption characteristics of tissues, and traditional methods have difficulty in accurately capturing subtle changes in these light signals; therefore, only the prediction results based on deep learning methods are presented in the results.

On the PURE dataset, it can be observed from the overall trend that the performance of multi-task methods was far better than that of single-task methods; meanwhile, on the VIPL-HR dataset, the test results of rSPO were better than ResNet18 in every indicator. This is because the scenarios in the PURE dataset are relatively simple, and multi-task learning can better capture the correlations between different physiological signals and make full use of them to improve the performance of the model. In contrast, the data in the VIPL-HR dataset are more complex, contain a variety of changing factors, and have higher requirements for the generalizability of the model. ResNet18, as a classic CNN structure, needs to handle multiple tasks simultaneously, and it is difficult to find the best balance between different tasks; meanwhile, rSPO—as a method specifically designed to estimate SpO2—constructs a CNN model that considers DC and AC components, effectively extracts features related to SpO2 through the extraction and fusion of components from the spatiotemporal map, and can better meet the requirements of the VIPL-HR data set for the SpO2 estimation task [64].

In the PURE dataset, the performance of our proposed MultiPhys was better than that of all other multi-task deep learning models. The MAE indicator was about 46% higher than that of the worst-performing ResNet18 model and about 25% higher than the best-performing PhysMLE-T model. As mentioned in the context of HR estimation, our MultiPhys optimizes the processing of visual tasks and adopts a hierarchical structure, as well as performing feature selection after feature extraction, thus increasing the ability to capture skin light cycle changes, and can also more specifically select features related to SpO2.

#### 4.4.3. RR Estimation

In the RR measurement task, as shown in Table 4, only the V4V dataset was used. The other two datasets either do not provide RR-related labels or have inaccurate labels. In the RR measurement model, except for ARM-SpO2 [61] and rSPO [64], the rest of the data were estimated using the deep learning-based multi-task measurement models. Compared to the traditional ARM-SpO2 method, the deep learning method overall reduced the MAE indicator by about one-fifth, as the traditional method has limitations in dealing with complex environments and variable movements, and cannot fully utilize large training datasets to learn information-related features.

In the single-task deep learning method, the MAE indicator of rSPO was about 9% ahead of ResNet18, the RMSE indicator was 3% ahead, and the P was 33% ahead. This shows that, as a classic CNN model, ResNet18’s measurement performance for a certain physiological indicator will decrease in the context of simultaneously measuring multiple physiological indicators. We developed MultiPhys precisely in order to address this seesaw effect.

However, compared to PhysMLE-T, our proposal did not show a significant advantage in the RMSE and P indicators, which may be as PhysMLE utilizes some physiological prior knowledge and adopts a more complex loss function and hyperparameters for training, thus improving its RR prediction ability [47]; as such, it can be seen that our MultiPhys still has room for improvement in RR prediction.

### 4.5. Cross-Dataset Estimation

To evaluate the generalization ability of MultiPhys, we carried out a cross-dataset test on the VIPL-HR and PURE datasets [57]; in particular, we first trained the model on the VIPL-HR dataset and then tested it on the PURE dataset. The results are shown in Table 5.

From the results, it can be observed that the generalization ability of our proposed model was not always the best. For example, in the HR and SpO2 measurement tasks, the best indicators were achieved by PhysMLE-R and PhysMLE-T, respectively. This is because our MultiPhys model is not optimized for generalization. In addition, in the HR test, although there was still some gap in the training results compared to the rPPG-MAE [62] and Contrast-Phys+ [63] models, which have been pre-trained with a large amount of data, as a multi-task model, it has the advantage of being broader and more comprehensive in predicting physiological indicators. Compared to the rPPG-MAE and Contrast-Phys+ models, our MultiPhys can measure not only HR but also SpO2, and its measurement effect is far better than that of both traditional and deep learning-based single-task measurement methods.

### 4.6. Impact of the Number of Layers

Although the performance of our MultiPhys model in measuring HR did not exceed that of the pre-trained models, it was very close. This is because our model can use the correlations between different physiological indicators for training, and these correlations are universal. For example, heart rate and blood oxygen indicators between people of different skin colors can be measured by identifying changes in light absorption of facial skin [70], and this correlation will not be affected by physiological factors such as the color of the skin or the gender of the person. This is why our model, although it was not pre-trained, achieved comparable predictive power to the pre-trained models in the cross-dataset measurement test.

**Figure 4:**
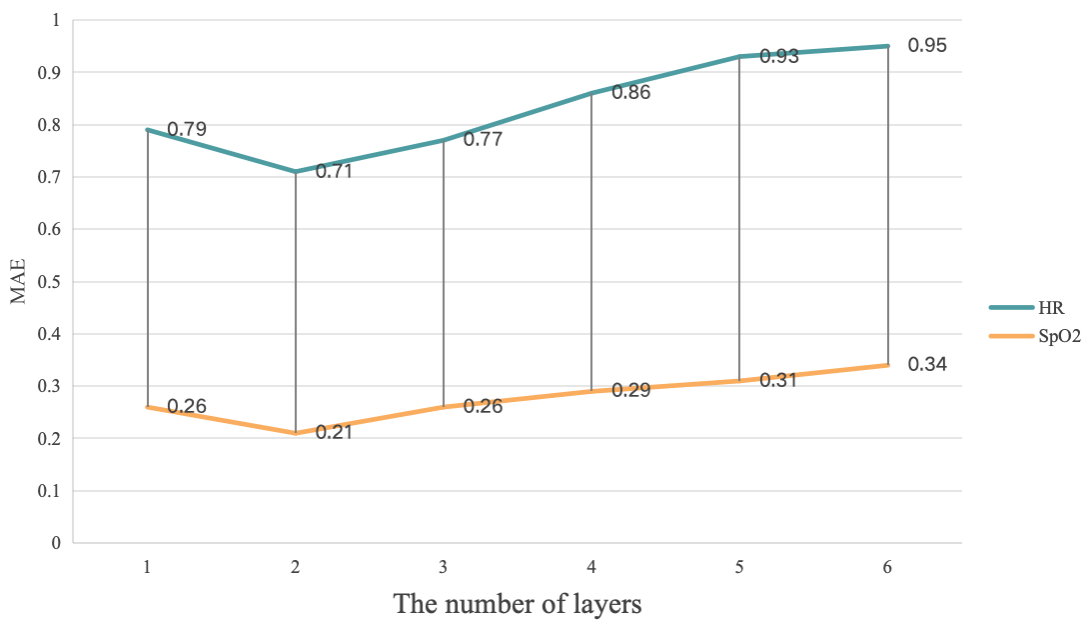
Impact of layers test based on **PURE** dataset.

**Figure 5:**
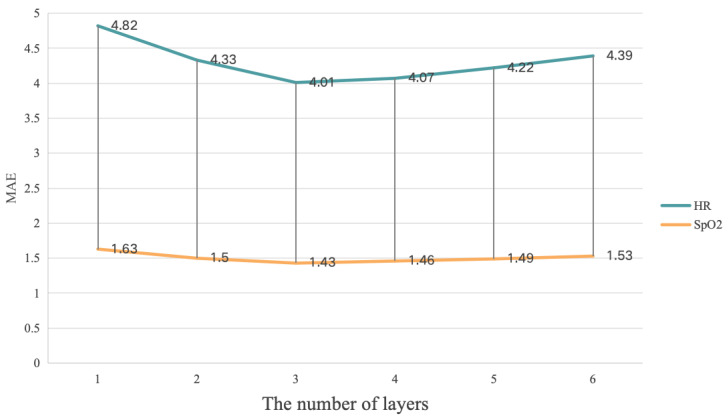
Impact of layers test based on **VIPL-HR** dataset.

**Figure 6:**
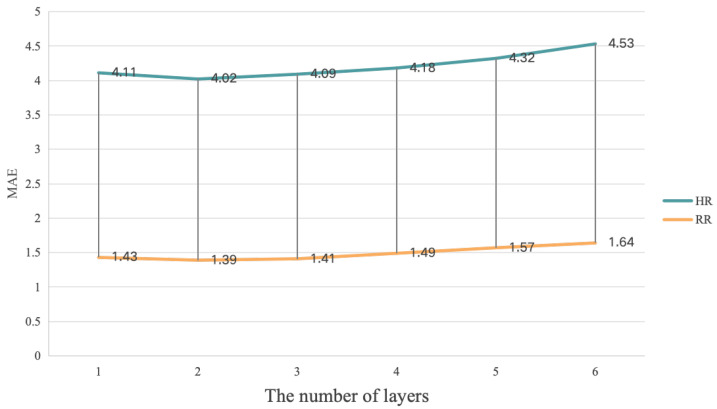
Impact of layers test based on **V4V** dataset.

We conducted ablation experiments on the different number of layers (*N*) in the MultiPhys blocks of our proposal on the PURE, VIPL-HR, and V4V datasets. In particular, we adjusted *N* from 1 to 6, and the obtained MAE values are presented in Figure 4, Figure 5, and Figure 6, respectively.

In Figure 4 and Figure 6, it can be seen that the MultiPhys blocks with 2 layers had the best HR, SpO2, and RR estimation performance while, in Figure 5, the MultiPhys blocks with 3 layers had the best HR and SpO2 estimation performance. The reason for this phenomenon may be that the VIPL-HR dataset, compared to the other two datasets, has more complex lighting conditions and head movements [71] and, so, learning more complex and abstract representations of features in more layers may result in better prediction performance. From the overall trend of our three figures, we can observe that, when *N* increases from 1 to 2 or 3, the MAE decreases while, when it gradually increases from 2 or 3, the MAE will gradually increase. This is because, as *N* increases, our MultiPhys blocks can automatically learn multi-level features from the original physiological data and construct more complex nonlinear functions, thereby better fitting the data. However, when *N* reaches 2 or 3 and continues to increase, it can cause the model to learn the noise and irrelevant features in the training data, thereby overfitting the training data, and there may also be cases of disappearance or explosion of the gradient, which makes the model difficult to train and reduces the performance of the model. In conclusion, in our experiment, we chose N=2 as the optimal number of layers for MultiPhys blocks.

## 5. Conclusions

In this study, we proposed a multi-task remote physiological measurement model called MultiPhys. In terms of its architecture, this model combines the advantages of CNN, Transformer, and Mamba architectures. Specifically, compared with the traditional CNN and Transformer architectures, our model simultaneously reduces the computational complexity and acquires the ability to capture global context and long-distance dependencies, enabling more efficient extraction of shared features. In addition, a gating mechanism is added, which can perform more targeted feature selection on the task-shared representation, thus allowing relevant features to be selected for each physiological indicator. To evaluate the feasibility of our method, we tested the model on three datasets, and the results indicated that MultiPhys performs better than other previous models. Nevertheless, our model still has room for improvement. In the cross-dataset and hyper-parameter sensitivity tests, compared with some methods in the cross-dataset test, our model did not always perform the best. In the future, we should train the model on larger-scale datasets and conduct larger-scale generalization tests [72,73]. Furthermore, in terms of the computational cost, a lighter fusion model should be developed considering the needs of real-time estimation in the real world.

## Figures and Tables

**Table 1:** A summary of symbols and descriptions.

Symbol	Description
X	The input of MultiPhys.
$\hat{Y}$	The output of MultiPhys.
$\hat{y}$	The indicator’s prediction.
H,W,C	Height, width, and channels.
*N*	The number of layers.
α	The output of the previous CNN layer, serving as the input of the next CNN layer.
A,B,C	Evolution matrix, projection matrix, and projection matrix.
$\bar{A},\bar{B},\bar{C}$	Discrete evolution matrix, discrete projection matrix, and discrete projection matrix.
exp	Exponential function.
ψ	The learnable hidden state.
ρ	The input of SSM.
φ	The output of SSM.
$\bar{K}$	Discrete structured convolution kernel.
Δ	Time-scale.
*I*	Identity matrix.
*S*	The sequence length.
β	The output of the path.
δ	The output of Conv Blocks.
γ	The input of the MLP layer.
τ	The number of MLP layers.
Γ	Softmax Function.
∗	Convolution operation.
Q,K,V	Query, key, and value.
*h*	The number of attention heads.
σ	The output of the gate.
*r*	Task-shared Representation.
$r^{\prime }$	Task-specific feature.
θ	Adaption parameters.
$w_{i}$	Trade-off parameters.
*L*	Loss function.

**Table 2:** Experimental data based on the PURE dataset.

		PURE					
			**HR**			**SpO2**	
**Method Type**	**Method**	**MAE ↓**	**RMSE ↓**	**P ↑**	**MAE ↓**	**RMSE ↓**	**P ↑**
**Single-task** **Traditional**	**GREEN [49]**	10.32	14.27	0.52	—	—	—
	**CHROM [24]**	9.79	12.76	0.37	—	—	—
	**POS [60]**	9.82	13.44	0.34	—	—	—
	**ARM-SpO2 [61]**	—	—	—	2.25	2.51	0.20
**Single-task** **Deep**	**RhythmNet [57]**	7.89	10.66	0.62	—	—	—
	**BVPNet [16]**	7.44	10.51	0.64	—	—	—
	**Dual-GAN [59]**	7.65	10.86	0.63	—	—	—
	**PhysFormer++ [21]**	7.16	10.14	0.65	—	—	—
	**EfficientPhys [37]**	8.21	11.30	0.57	—	—	—
	**rPPG-MAE [62]**	1.41	1.73	0.98	—	—	—
	**Contrast-Phys+ [63]**	1.28	1.98	0.98	—	—	—
	**rSPO [64]**	—	—	—	1.17	1.40	0.48
**Multi-task** **Deep**	**ResNet18 [65]**	1.29	1.29	**0.99**	0.39	0.52	0.96
	**ViT-base [66]**	1.34	1.39	**0.99**	0.31	0.57	0.98
	**PhysMLE-R [47]**	0.81	1.24	**0.99**	0.34	0.41	0.98
	**PhysMLE-T [47]**	1.00	1.53	**0.99**	0.28	0.36	0.99
	**MultiPhys**	**0.71**	**1.11**	**0.99**	**0.21**	**0.30**	**0.99**

Note: In this and the following tables, bold text indicates the best result within each column. The ↓ symbol indicates that the smaller the indicator, the better the model performance. And the ↑ symbol indicates that the larger the indicator, the better the model performance.

**Table 3:** Experimental data based on the VIPL-HR dataset.

		VIPL-HR					
			**HR**			**SpO2**	
**Method Type**	**Method**	**MAE ↓**	**RMSE ↓**	**P ↑**	**MAE ↓**	**RMSE ↓**	**P ↑**
**Single-task** **Traditional**	**GREEN [49]**	12.18	18.23	0.25	—	—	—
	**CHROM [24]**	11.44	16.97	0.28	—	—	—
	**POS [60]**	14.59	21.26	0.19	—	—	—
	**ARM-SpO2 [61]**	—	—	—	8.11	12.23	0.08
**Single-task** **Deep**	**RhythmNet [57]**	5.30	8.14	0.76	—	—	—
	**BVPNet [16]**	5.34	7.85	0.70	—	—	—
	**Dual-GAN [59]**	4.93	7.68	0.81	—	—	—
	**PhysFormer++ [21]**	4.88	7.62	0.80	—	—	—
	**EfficientPhys [37]**	5.84	8.91	0.72	—	—	—
	**rPPG-MAE [62]**	4.82	7.56	0.78	—	—	—
	**Contrast-Phys+ [63]**	4.64	7.41	0.79	—	—	—
	**rSPO [64]**	—	—	—	2.36	6.54	0.16
**Multi-task** **Deep**	**ResNet18 [65]**	4.64	7.98	0.80	2.59	6.77	0.12
	**ViT-base [66]**	4.91	8.57	0.76	1.64	5.78	0.19
	**PhysMLE-R [47]**	4.29	7.37	0.81	1.85	5.92	0.17
	**PhysMLE-T [47]**	4.79	8.06	0.79	1.55	5.40	0.21
	**MultiPhys**	**4.01**	**7.17**	**0.82**	**1.43**	**5.28**	**0.22**

Note: In this and the following tables, **bold** text indicates the best result within each column. The ↓ symbol indicates that the smaller the indicator, the better the model performance. And the ↑ symbol indicates that the larger the indicator, the better the model performance.

**Table 4:** Experimental data based on the V4V dataset.

		V4V					
			**HR**			**RR**	
**Method Type**	**Method**	**MAE ↓**	**RMSE ↓**	**P ↑**	**MAE ↓**	**RMSE ↓**	**P ↑**
**Single-task** **Traditional**	**GREEN [49]**	12.18	18.23	0.25	—	—	—
	**CHROM [24]**	11.44	16.97	0.28	—	—	—
	**POS [60]**	14.59	21.26	0.19	—	—	—
	**ARM-RR [61]**	—	—	—	8.11	12.23	0.08
**Single-task** **Deep**	**RhythmNet [57]**	5.30	8.14	0.76	—	—	—
	**BVPNet [16]**	5.34	7.85	0.70	—	—	—
	**Dual-GAN [59]**	4.93	7.68	0.81	—	—	—
	**PhysFormer++ [21]**	4.88	7.62	0.80	—	—	—
	**EfficientPhys [37]**	5.84	8.91	0.72	—	—	—
	**rPPG-MAE [62]**	4.93	7.96	0.80	—	—	—
	**Contrast-Phys+ [63]**	4.41	8.01	0.79	—	—	—
	**rSPO [64]**	—	—	—	2.36	6.54	0.16
**Multi-task** **Deep**	**ResNet18 [65]**	4.64	7.98	0.80	2.59	6.77	0.12
	**ViT-base [66]**	4.91	8.57	0.76	1.64	5.78	0.19
	**PhysMLE-R [47]**	4.29	7.37	0.81	1.85	5.92	0.17
	**PhysMLE-T [47]**	4.79	8.06	0.79	1.55	**5.40**	**0.21**
	**MultiPhys**	**4.02**	**7.11**	**0.82**	**1.39**	5.41	**0.21**

Note: In this and the following tables, **bold** text indicates the best result within each column. The ↓ symbol indicates that the smaller the indicator, the better the model performance. And the ↑ symbol indicates that the larger the indicator, the better the model performance.

**Table 5:** Cross-dataset experiment of training on the VIPL-HR and testing on the PURE.

		VIPL-HR→PURE					
			**HR**			**SpO2**	
**Method Type**	**Method**	**MAE ↓**	**RMSE ↓**	**P ↑**	**MAE ↓**	**RMSE ↓**	**P ↑**
**Single-task** **Traditional**	**GREEN [49]**	10.32	14.27	0.52	—	—	—
	**CHROM [24]**	9.79	12.76	0.37	—	—	—
	**POS [60]**	9.82	13.44	0.34	—	—	—
	**ARM-SpO2 [61]**	—	—	—	2.25	2.51	0.20
**Single-task** **Deep**	**RhythmNet [57]**	2.99	3.65	0.85	—	—	—
	**BVPNet [16]**	2.94	3.56	0.85	—	—	—
	**Dual-GAN [59]**	2.63	3.32	0.86	—	—	—
	**PhysFormer++ [21]**	2.14	3.11	0.88	—	—	—
	**EfficientPhys [37]**	3.11	3.87	0.83	—	—	—
	**rPPG-MAE [62]**	2.10	3.03	0.88	—	—	—
	**Contrast-Phys+ [63]**	2.01	2.94	0.89	—	—	—
	**rSPO [64]**	—	—	—	2.10	1.99	0.31
**Multi-task** **Deep**	**RestNet18 [65]**	2.33	3.34	0.86	1.85	1.75	0.52
	**ViT-base [66]**	2.59	3.50	0.85	1.70	1.58	0.58
	**PhysMLE-R [47]**	**1.81**	**2.76**	**0.91**	1.49	1.38	0.62
	**PhysMLE-T [47]**	2.02	3.01	0.89	**1.40**	**1.25**	**0.64**
	**MultiPhys**	2.08	3.11	0.89	1.48	1.35	0.62

Note: In this and the following tables, **bold** text indicates the best result within each column. The ↓ symbol indicates that the smaller the indicator, the better the model performance. And the ↑ symbol indicates that the larger the indicator, the better the model performance.

## Data Availability

The PURE, VIPL-HR, and V4V datasets used in this study can be accessed at https://www.tu-ilmenau.de/universitaet/fakultaeten/fakultaet-informatik-und-automatisierung/profil/institute-und-fachgebiete/institut-fuer-technische-informatik-und-ingenieurinformatik/fachgebiet-neuroinformatik-und-kognitive-robotik/data-sets-code/pulse-rate-detection-dataset-pure, https://github.com/MayYoY/RhythmNet, and https://github.com/OpenXT/v4v, respectively. We accessed on 6 June 2024.

## Abbreviations

The following abbreviations are used in this manuscript:

rPPG	Remote Photoplethysmography
CNN	Convolutional Neural Network
BVP	Blood Volume Pulse
HR	Heart Rate
HRV	Heart Rate Variability
RR	Respiration Rate
SpO2	Blood oxygen Saturation
ECG	Electrocardiography
PPG	Photoplethysmography
SOTA	State-of-the-art
STMap	Spatial–temporal map
S4	Structured state space model
SSM	Structured State Space Model
